# Supplementary material for: When ecological marginality is not geographically peripheral: exploring genetic predictions of the centre-periphery hypothesis in the endemic plant Lilium pomponium
Source: PeerJ. 2021 Mar 10;9:e11039. doi: 10.7717/peerj.11039 (PMC7955672; doi:10.7717/peerj.11039)
Supplement: Supplemental Information 1 — Blue circles are studied populations, circle size shows the distance from the geographical center. BIO1 = Annual Mean Temperature; BIO2 = mean diurnal range; BIO3 = isothermality (BIO2/BIO7); BIO4 = temperature seasonality; BIO5 = max temperature of warmest month; BIO6 = min temperature of coldest month; BIO7 = temperature annual range (BIO5-BIO6); BIO8 = mean temperature of wettest quarter; BIO9 = mean temperature of Driest quarter; BIO10 = mean temperature of warmest quarter; BIO11 = mean temperature of coldest quarter; BIO12 = annual precipitation; BIO13 = precipitation of wettest month; BIO14 = precipitation of driest month; BIO15 = precipitation seasonality; BIO16 = precipitation of wettest quarter; BIO17 = precipitation of driest quarter; BIO18 = precipitation of warmest quarter; BIO19 = precipitation of coldest quarter. [file peerj-09-11039-s001.pdf]

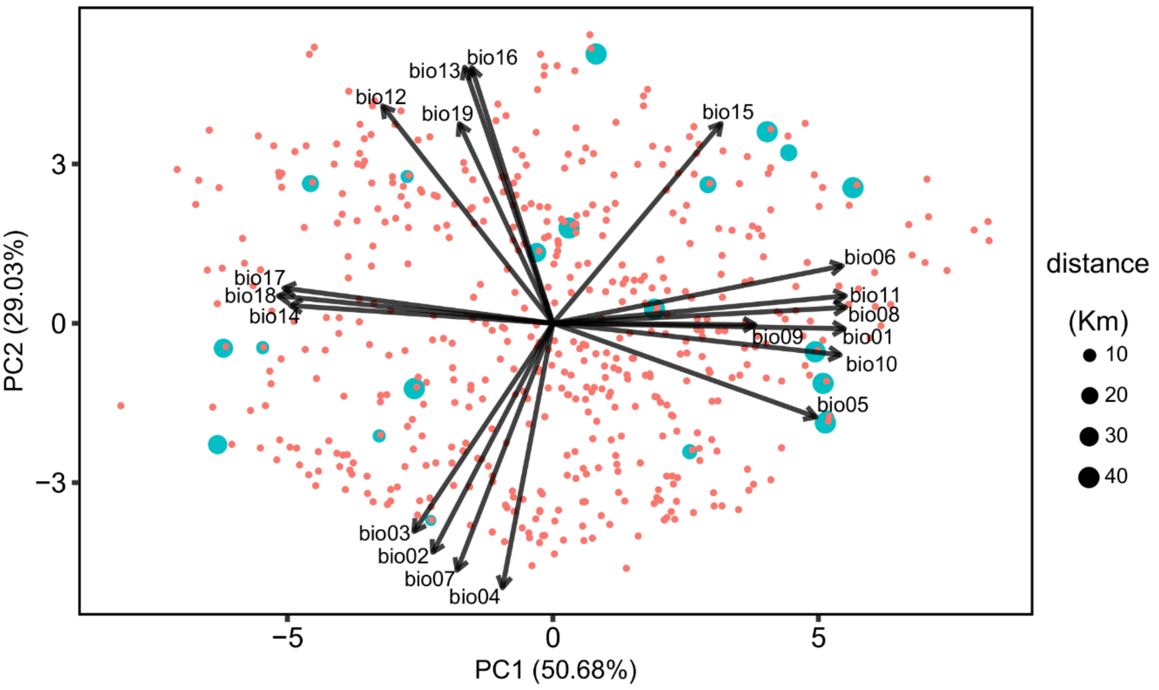

Figure S1. Principal component analyses based on bioclimatic variables for 881 occurrences of *Lilium pomponium*. Blue circles are studied populations, circle size shows the distance from the geographical centre. BIO1 = Annual Mean Temperature; BIO2 = mean diurnal range; BIO3 = isothermality (BIO2/BIO7); BIO4 = temperature seasonality; BIO5 = max temperature of warmest month; BIO6 = min temperature of coldest month; BIO7 = temperature annual range (BIO5-BIO6); BIO8 = mean temperature of wettest quarter; BIO9 = mean temperature of Driest quarter; BIO10 = mean temperature of warmest quarter; BIO11 = mean temperature of coldest quarter; BIO12 = annual precipitation; BIO13 = precipitation of wettest month; BIO14 = precipitation of driest month; BIO15 = precipitation seasonality; BIO16 = precipitation of wettest quarter; BIO17 = precipitation of driest quarter; BIO18 = precipitation of warmest quarter; BIO19 = precipitation of coldest quarter.
